# Glycosaminoglycans Regulate CXCR3 Ligands at Distinct Levels: Protection against Processing by Dipeptidyl Peptidase IV/CD26 and Interference with Receptor Signaling

**DOI:** 10.3390/ijms18071513

**Published:** 2017-07-13

**Authors:** Mieke Metzemaekers, Anneleen Mortier, Rik Janssens, Daiane Boff, Lotte Vanbrabant, Nicole Lamoen, Jo Van Damme, Mauro M. Teixeira, Ingrid De Meester, Flávio A. Amaral, Paul Proost

**Affiliations:** 1 Laboratory of Molecular Immunology, Department of Microbiology and Immunology, Rega Institute for Medical Research, KU Leuven, Herestraat 49 box 1042, B-3000 Leuven, Belgium; mieke.metzemaekers@kuleuven.be (M.M.); anneleen_mortier@hotmail.com (A.M.); rik.janssens@kuleuven.be (R.J.); daianebff@gmail.com (D.B.); lotte.vanbrabant@kuleuven.be (L.V.); jo.vandamme@kuleuven.be (J.V.D.); 2 Imunofarmacologia, Departamento de Bioquímica e Imunologia, Instituto de Ciências Biológicas, Universidade Federal de Minas Gerais, Av Antonio Carlos 6627, Pampulha, Belo Horizonte 31270-901, Minas Gerais, Brazil; mmtex.ufmg@gmail.com (M.M.T); dr.famaral@gmail.com (F.A.A.); 3 Laboratory of Medical Biochemistry, Department of Pharmaceutical Sciences, University of Antwerp, Universiteitsplein 1 S6, Wilrijk B-2610, Belgium; ingrid.demeester@uantwerpen.be

**Keywords:** chemokine, glycosaminoglycan, leukocyte migration, posttranslational modification, CXCR3, dipeptidyl peptidase IV, CD26

## Abstract

CXC chemokine ligand (CXCL)9, CXCL10 and CXCL11 direct chemotaxis of mainly T cells and NK cells through activation of their common CXC chemokine receptor (CXCR)3. They are inactivated upon NH_2_-terminal cleavage by dipeptidyl peptidase IV/CD26. In the present study, we found that different glycosaminoglycans (GAGs) protect the CXCR3 ligands against proteolytic processing by CD26 without directly affecting the enzymatic activity of CD26. In addition, GAGs were shown to interfere with chemokine-induced CXCR3 signaling. The observation that heparan sulfate did not, and heparin only moderately, altered CXCL10-induced T cell chemotaxis in vitro may be explained by a combination of protection against proteolytic inactivation and altered receptor interaction as observed in calcium assays. No effect of CD26 inhibition was found on CXCL10-induced chemotaxis in vitro. However, treatment of mice with the CD26 inhibitor sitagliptin resulted in an enhanced CXCL10-induced lymphocyte influx into the joint. This study reveals a dual role for GAGs in modulating the biological activity of CXCR3 ligands. GAGs protect the chemokines from proteolytic cleavage but also directly interfere with chemokine–CXCR3 signaling. These data support the hypothesis that both GAGs and CD26 affect the in vivo chemokine function.

## 1. Introduction

The family of chemotactic cytokines or chemokines is constituted by a group of low molecular mass proteins that direct specific leukocyte migration in a time- and site-dependent manner in health and disease [1,2]. Chemokines are not only crucial players in basal innate and adaptive immune mechanisms, but are also implicated in a broad range of additional physiological and pathophysiological processes ranging from embryonic development and angiogenesis to cancer and autoimmune diseases [3,4,5,6,7,8,9]. According to the localization of the conserved NH_2_-terminal cysteine residues, a sub classification into CXC, CC, CX_3_C and C chemokine subfamilies is respected. CXCL9, CXCL10 and CXCL11 are three CXC chemokines that lack a conserved Glu-Leu-Arg (ELR) amino acid motif and exert their chemotactic activity through interaction with their common G protein-coupled receptor (GPCR) CXCR3 [10,11]. In order of increasing potency, CXCL9, CXCL10 and CXCL11 activate CXCR3, which is strongly expressed on type-1 helper (Th1) CD4+ T cells, effector CD8+ T cells and certain innate leukocytes including natural killer (NK) cells [10,11,12]. In addition to their chemotactic effects, angiostatic properties have been attributed to the CXCR3 ligands [3,5,11,13]. Unique characteristics have been claimed to individual CXCR3 ligands. For example, CXCL11 is the only CXCR3 ligand that also interacts with the atypical chemokine receptor ACKR3 (also known as CXCR7) [14,15,16]. CXCL9 contains a unique COOH-terminal tail that consists for about 50% of basic amino acids and differs not only from the two other CXCR3 ligands, but also from almost all chemokines in general (vide infra) [1,17].

As suggested by their alternative names, being monokine induced by interferon (IFN)-γ (Mig), IFN-γ-inducible protein of 10 kDa (IP-10) and IFN-γ-inducible T cell α chemoattractant (I-TAC), for CXCL9, CXCL10 and CXCL11 respectively, these are inflammatory chemokines with IFN-γ as a major inducer. However, the specific expression of individual CXCR3 ligands is differently regulated. Induction of CXCL9 expression is truly IFN-γ dependent [18,19], whereas expression of CXCL10 is also induced by a variety of innate stimuli including IFN-α or IFN-β [18,20]. IFN-γ and IFN-β, but not IFN-α, are potent stimulators of CXCL11 expression [21]. Consequently, despite their mutual structural similarities and shared, unique signaling receptor, the CXCR3 ligands show a significant degree of redundancy only in vitro that seems less the case in vivo [10]. During the course of immune responses, the temporal and spatial expression patterns of individual CXCR3 ligands are ligand-specific, with each CXCR3 ligand being regulated by different stimuli and expressed by different cell types [10,22,23]. It is therefore believed that in vivo, CXCL9, CXCL10 and CXCL11 each play a unique role in fine-tuning the trafficking of T cells. Accordingly, in certain inflammatory in vivo models, deficiency for one specific CXCR3 ligand cannot be countervailed by the presence of the two others [24,25,26,27]. Additionally, in vivo models exist where full T cell infiltration requires cooperation between the CXCR3 ligands [28,29]. To add even more complexity to the CXCR3-chemokine loop, apparent ligand antagonism has been described between CXCR3 agonists [30]. Furthermore, one has to keep in mind that most studies in mice concern CXCL10, whereas CXCL9 and certainly CXCL11 have been studied to a lesser extend in vivo. Moreover, it has been reported that a frame shift causing the presence of a stop codon in the CXCL11 gene thus, resulting in deficiency for CXCL11 is present in one of the routinely used mouse strains, i.e., C57BL6 mice [31].

In addition to interaction with chemokine receptors, chemokine-induced leukocyte migration in vivo usually requires also interaction between chemokines and GAGs [32,33,34,35,36,37,38,39]. GAGs, e.g., heparin, heparan sulfate, chondroitin sulfate, keratan sulfate, dermatan sulfate and hyaluronic acid, are present in the extracellular matrix and at the cell surface, usually as part of proteoglycan structures (GAG chains attached to a protein core). Due to their sulfate groups, these heterogeneous polysaccharides are negatively charged and thus attractive interaction partners for the highly basic chemokines [40]. GAGs retain chemokines on endothelial surfaces and prevent washout of chemokines by the blood flow [33]. Of the three main CXCR3 agonists, CXCL9 in particular is an extremely efficient GAG interacting chemokine, due to its highly positively charged long COOH-terminal tail that consists for circa 50% of basic amino acids. To investigate the function of the COOH-terminal region of CXCL9, our lab previously synthesized several peptides, derived from the COOH-terminal region of CXCL9 [17,41]. Specifically, the longest peptide CXCL9 (74–103) was found to compete with CXCL8 for GAG-binding, thereby preventing CXCL8 from performing its neutrophil-chemotactic function in vivo [17]. CXCL8 is an ELR positive CXC chemokine that activates CXCR1 and CXCR2 and is considered the main human neutrophil-attracting chemokine [4,42,43]. In general, binding to GAGs facilitates chemokine retention and assists in generation of a chemokine gradient that directs leukocyte migration in vivo [33,44]. Interaction with GAGs also mediates chemokine oligomerization [40] and is thought to play a role in either cis or trans (on endothelial cells) presentation of chemokines to their receptors [45]. In addition, GAGs were shown to serve as a protective factor that prevent the chemokines CXCL12 and CCL11 from proteolysis by specific enzymes [46,47].

Since the identification of chemokines, it has been evidenced that the biological availability and functioning of chemokines is coordinated at multiple levels. These include alternative splicing and mutual synergistic or antagonistic effects between certain chemokines, in addition to the aforementioned interactions with GAGs, specific GPCRs and atypical chemokine receptors [44]. Moreover, a major role for posttranslational processing, e.g., proteolysis, citrullination, glycosylation and nitration, has been recognized in fine-tuning the exact chemokine function and receptor specificity in vitro and in vivo [44,48,49,50,51]. An enzyme that has been shown to provoke NH_2_-terminal processing of various chemokines including CXCL9, CXCL10 and CXCL11 is dipeptidyl peptidase IV or CD26 [50,52,53]. In addition to its enzymatic activity as a serine protease, the multifunctional or “moonlighting” protein CD26 functions as a receptor, costimulator for T cell activation, adhesion molecule and has been associated with apoptosis [54,55,56,57]. The membrane-bound enzyme is expressed on cells of different origins, including certain immune cells, whereas soluble proteolytically active CD26 exists in several body fluids such as plasma and seminal fluid. CD26 preferentially removes the two most NH_2_-terminal amino acids from substrates whose penultimate position is occupied by a (hydroxy) proline or alanine residue. Pro is present at this position in a number of chemokine sequences. The NH_2_-terminal chemokine domain is responsible for GPCR binding and activation and, consequently, limited proteolysis by CD26 (but also by other enzymes) may have drastic effects on the biological functioning of a chemokine [50,51,52]. It turned out that the biological effect of CD26-mediated cleavage is highly complex and depends on the chemokine ligand involved. For all three CXCR3 agonists, it was previously demonstrated that processing by CD26 results in drastic loss of receptor signaling and impaired capacity to direct lymphocyte chemotaxis, while leaving the angiostatic properties of these chemokines unaffected [53]. For human CXCL10 and CXCL11, the corresponding CD26-truncated isoforms CXCL10 (3–77) and CXCL11 (3–73) were previously isolated from natural sources, including conditioned medium from MG-63-osteosarcoma cells, fibroblasts and keratinocytes [22,58,59,60,61].

In the present study, we wanted to provide new insights in the intriguing role of GAGs in the regulation of the activity of CXCR3 agonists. Specifically, we investigated the effects of GAGs on the CXCR3 chemokine dialog and on CD26-mediated processing of CXCR3 agonists. The relationship between GAGs, CD26, CXCR3 and its chemokine ligands was found to be highly complex and dual: while GAGs dose-dependently preserved CXCL9, CXCL10 and CXCL11 from cleavage by CD26, these negatively charged macromolecules also negatively affected their calcium mobilizing capacities through CXCR3.

## 2. Results

### 2.1. Soluble GAGs Protected CXCR3 Ligands against Truncation by Soluble CD26

**Figure 1:**
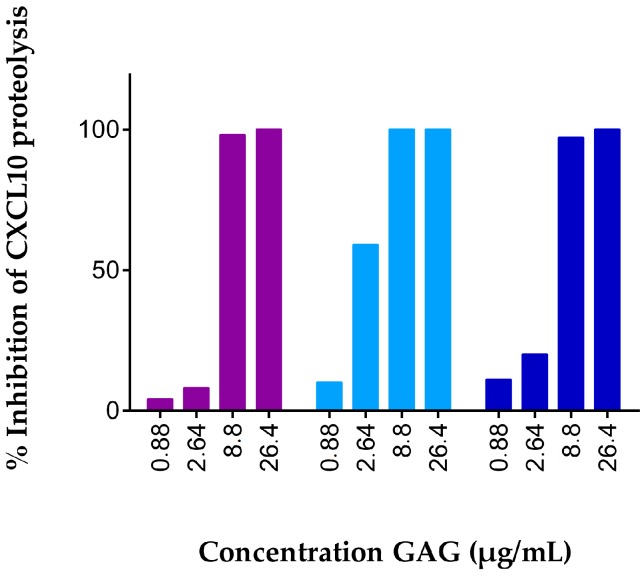
GAGs dose-dependently protect CXCL10 from cleavage by CD26. Recombinant human CXCL10 (20 µg/mL) was incubated with 12.5 U/L natural human CD26 and 0.88 to 26.4 µg/mL heparan sulfate (violet, ●), heparin DP30 (blue, ●), or heparin DP8 (deep blue, ●), in 50 mM Tris buffer supplemented with 1 mM EDTA (pH 7.5). Incubation of CXCL10 with CD26 in the absence of GAG was used as a control. Reactions were terminated after 2 h by acidification to 0.08% (*v*/*v*) trifluoroacetic acid (TFA). The ratio of truncated CXCL10 (3–77) over corresponding intact chemokine was determined with automated NH_2_-sequencing. Results are represented as percentages of GAG-mediated inhibition of proteolysis of CXCL10 by CD26.

GAGs were previously described to protect CXCL12 and CCL11 against proteolytic processing by specific enzymes, either directly or indirectly [46,47]. In the present study, we wondered whether this was also true for CD26-mediated truncation of the three most potent CXCR3 chemokine agonists and CD26 substrates CXCL9, CXCL10 and CXCL11. CXCL10, the most intensively studied CXCR3 ligand, was incubated for 2 h with 12.5 U/L natural human CD26 and final GAG concentrations up to 26.4 µg/mL. We reasoned that, following extraction of truncated and intact CXCL10 from incubation mixtures using C4 or strong cation exchange purification techniques, the percentage of CD26-mediated CXCL10-processing could be determined with mass spectrometry. However, using this protocol we failed to detect CXCL10 isoforms and we relied on automated NH_2_-terminal sequencing using Edman degradation for quantification of the CD26-mediated conversion of native CXCL10 towards CXCL10(3–77). Dose response experiments were conducted with heparan sulfate and the fixed chain-length heparin variants heparin DP30 and DP8. For all GAGs tested, CXCL10 was dose-dependently protected against proteolytic processing by CD26 (Figure 1). Incubation of CXCL10 with CD26 in the absence of GAG resulted in almost complete truncation of intact CXCL10 by two amino acids towards CXCL10 (3–77). At GAG concentrations up to 2.64 µg/mL, almost all CXCL10 was processed, whereas GAG concentrations of 8.8 µg/mL offered the chemokine almost full protection against proteolytic processing by the serine protease.

**Figure 2:**
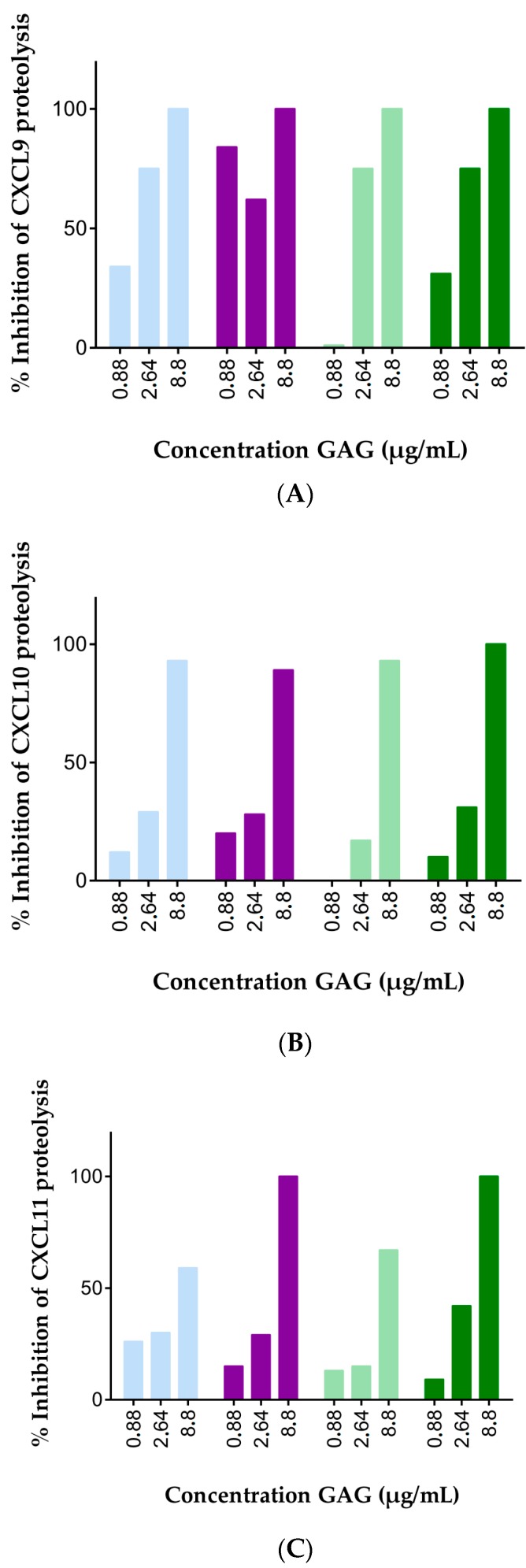
GAGs dose-dependently protect IFN-γ-inducible CXCR3 ligands from cleavage by CD26. Recombinant human: CXCL9 (**A**); CXCL10 (**B**) or CXCL11(**C**) (20 µg/mL) was incubated with 12.5 U/L natural human CD26 and 0.88 to 8.8 µg/mL heparan sulfate (violet, ●), heparin (light blue, ●), chondroitin sulfate A (light green, ●) or chondroitin sulfate C (green, ●) in 50 mM Tris buffer supplemented with 1 mM EDTA (pH 7,5). Reactions were terminated after 2 h by acidification to 0.08% (*v*/*v*) TFA. The ratio of truncated CXCL9(3–103), CXCL10(3–77) or CXCL11(3–73) over corresponding intact chemokines was determined after C18 purification and mass spectrometry. Results are represented as percentages of GAG-mediated inhibition of proteolysis of CXCL10 by CD26.

Inspired by these drastic effects of GAGs on CD26-mediated truncation of CXCL10, we investigated the effects of GAGs on CD26-mediated processing of the two other IFN-γ-inducible CXCR3 chemokine agonists, i.e., CXCL9 and CXCL11. Accordingly, we optimized the desalting process after the CD26 incubation and found that chemokines could be detected and quantified by mass spectrometry after pre-purification on C18 pipette tips if the GAG concentrations did not exceed 8.8 µg/mL. The three main CXCR3 agonists were incubated with CD26 in the absence or presence of 8.8, 2.64 or 0.88 µg/mL heparan sulfate, heparin, chondroitin sulfate A or chondroitin sulfate C. All these GAGs had a relative molecular mass (M_r_) of 40 kDa (data not shown). After an incubation period of 2 h, CD26 activity was stopped through acidification with TFA. Samples were desalted with C18 tips and subjected to mass spectrometry. Without GAGs, almost all CXCL10 and CXCL11 was cleaved by CD26 to CXCL10 (3–77) and CXCL11(3–73), respectively. In contrast, CXCL9 was processed by the enzyme for only about 20% (data not shown). These findings were in line with previous studies which demonstrated that CXCL10, and especially CXCL11, are highly efficient CD26 substrates, whereas the half-life of CXCL9 upon incubation with the enzyme is remarkably longer [53,62]. The presence of heparin, heparan sulfate, chondroitin sulfate A or chondroitin sulfate C in the incubation mixture protected the three IFN-induced CXCR3 ligands dose-dependently from truncation by CD26 (Figure 2, Figures S1–S3). At 8.8 µg/mL heparin, heparan sulfate, chondroitin sulfate A and C completely prevented processing of CXCL9 and CXCL10 by CD26. A comparable protective effect was obtained with heparan sulfate and chondroitin sulfate C, whereas heparin and chondroitin sulfate A were less efficient in protecting CXCL11 from proteolytic truncation by CD26. Thus, the obtained results indicated a dose-dependent, GAG-mediated chemokine protection against processing by CD26 for all GAGs tested. However, minor differences in efficiency were detected between the different GAGs although they all had a comparable molecular mass.

### 2.2. GAGs Did Not Inhibit the Enzymatic Activity of Soluble CD26 Directly

The exact enzymatic activity of the purified natural human CD26 sample was determined to be 4.6 ± 0.6 U/L (mean ± SEM, *n* = 3) using a chromogenic assay with Gly-Pro-p-nitroanilide (Gly-Pro-pNA) as the substrate. No pNA release was observed upon incubation of the substrate with the highest concentration of heparin DP30 in the absence of CD26. To investigate a direct effect of GAGs on the activity of the enzyme, the release of pNA was detected when Gly-Pro-pNA and CD26 were incubated in the absence or presence of 10 or 100 µg/mL heparin DP30. The CD26 activities in conditions with and without GAG were highly similar (Table 1). Thus, no evidence was found for GAGs to inhibit the proteolytic activity of CD26 directly, which was in line with a former study that reported that heparan sulfate did not inhibit the enzymatic activity of CD26 [46].

### 2.3. GAGs Interfered with Chemokine Signaling through CXCR3

**Figure 3:**
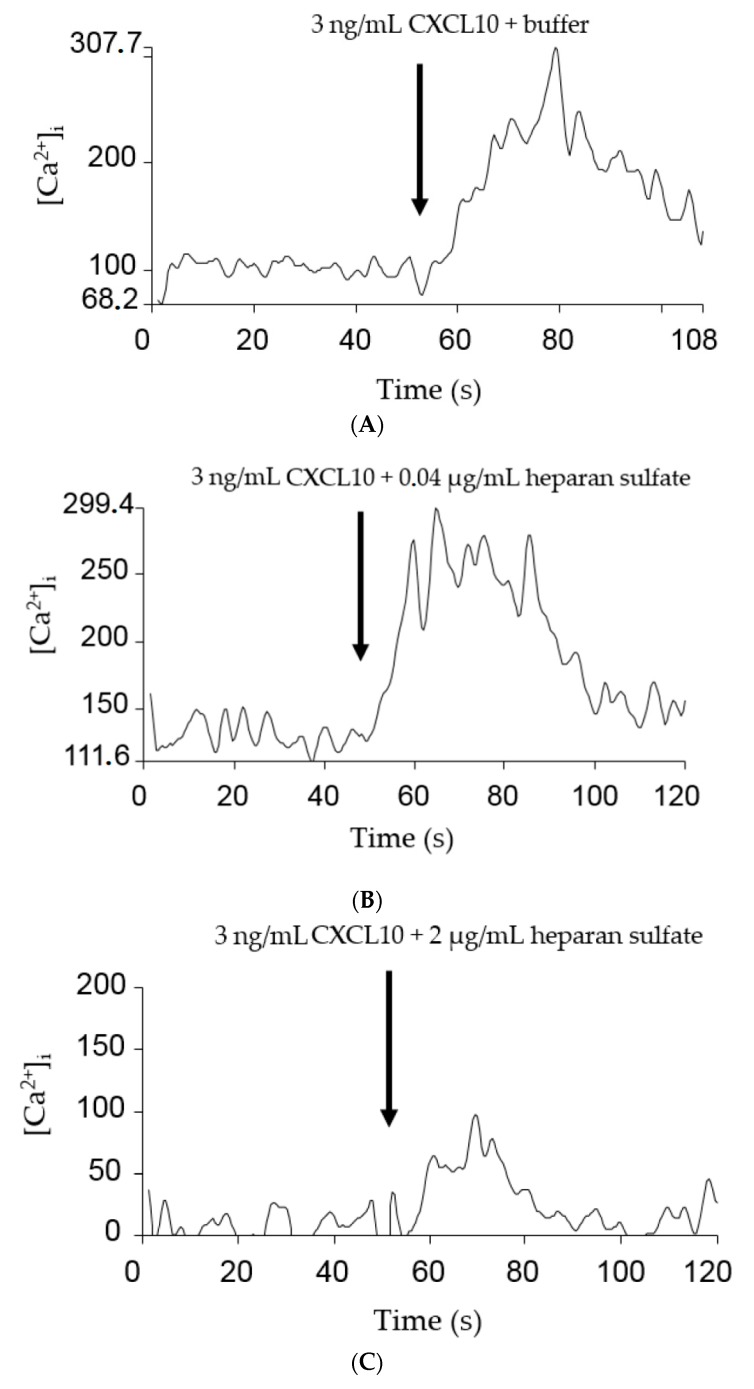

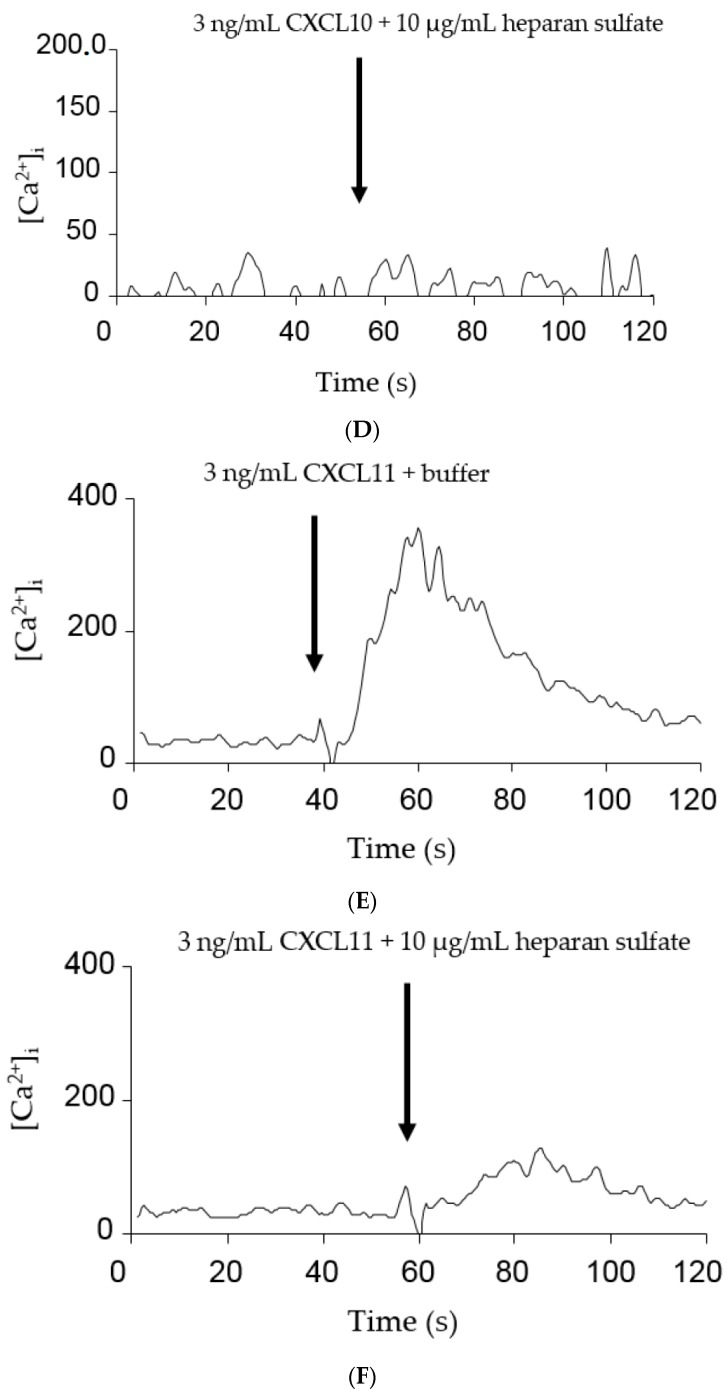

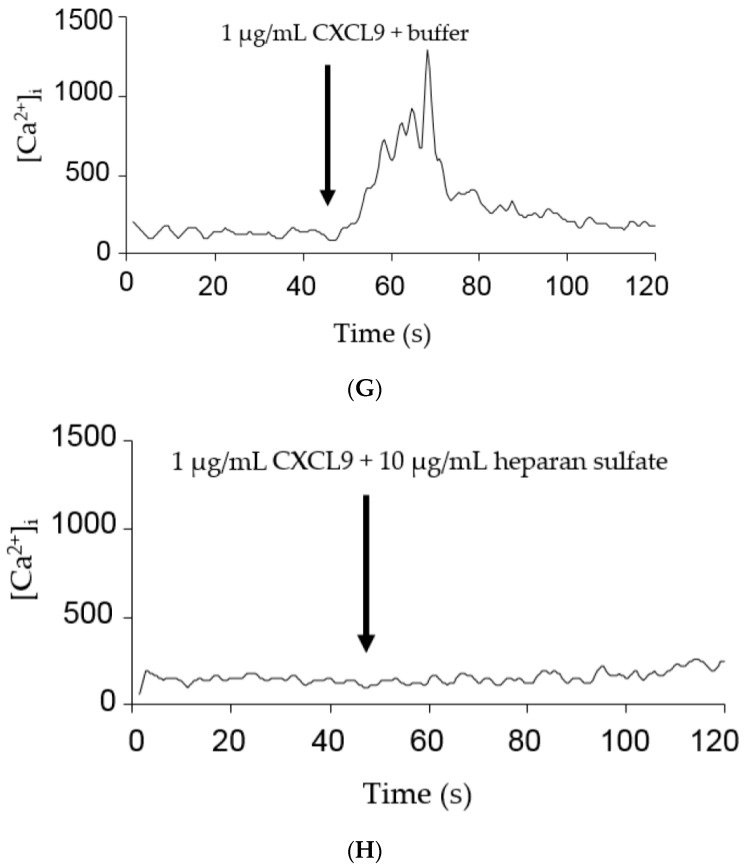
Effect of heparan sulfate on chemokine-induced calcium signaling through CXCR3. CHO/CXCR3A cells were stimulated with 3 ng/mL: CXCL10 (**A**–**D**); or CXCL11 (**E**,**F**); or 1 µg/mL CXCL9 (**G**,**H**) in the presence or absence of GAG. [Ca^2+^]_i_ concentrations were calculated using the equation of Grynkiewicz et al. Figures show representative experiments in which cells were simultaneously stimulated with chemokine and buffer (**A**,**E**,**G**); or 0.04 µg/mL (**B**); 2 µg/mL (**C**); or 10 µg/mL (**D**,**F**,**H**) heparan sulfate.

**Figure 4:**
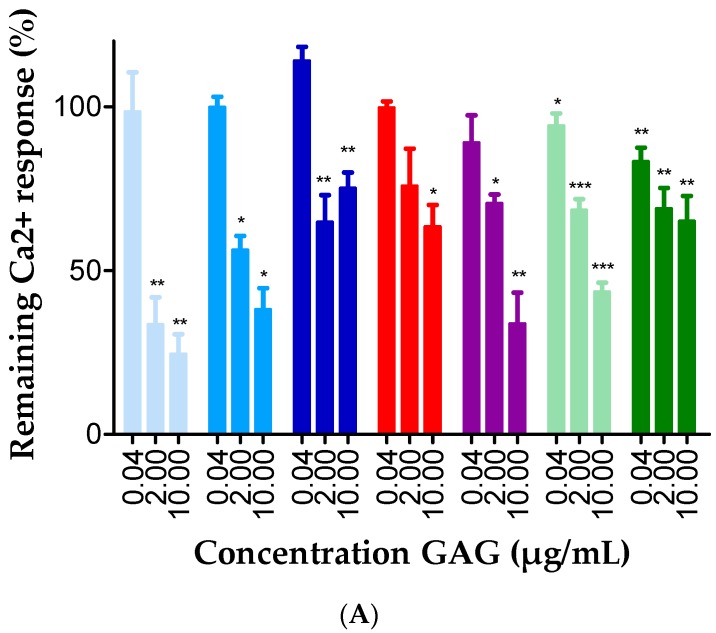

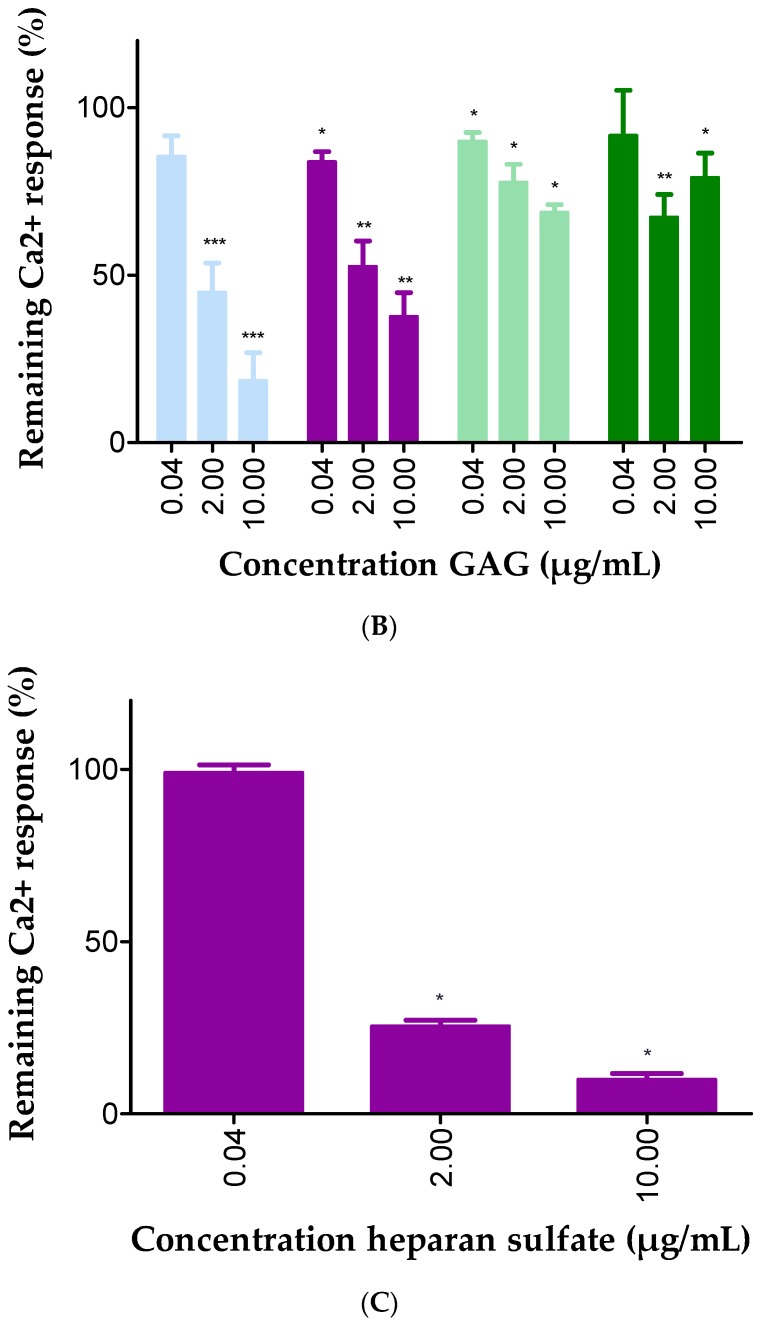
GAGs interfere with chemokine signaling through CXCR3. CHO/CXCR3A cells were stimulated with 3 ng/mL: CXCL10 (**A**); or CXCL11 (**B**); or 1 µg/mL CXCL9 (**C**) in the presence or absence of heparan sulfate (violet, ●), heparin (light blue, ●), heparin DP30 (blue, ●), heparin DP8 (deep blue, ●), dermatan sulfate (red, ●), chondroitin sulfate A (light green, ●) or chondroitin sulfate C (green, ●). [Ca^2+^]_i_ concentrations were calculated using the equation of Grynkiewicz et al. Mann–Whitney U-tests were performed to statistically compare [Ca^2+^]_i_ concentrations obtained after stimulation with CXCL9, CXCL10 or CXCL11 plus GAG, with [Ca^2+^]_i_ concentrations that resulted from stimulation with, respectively, CXCL9, CXCL10 or CXCL11 only (* = *p* < 0.05; ** = *p* < 0.01; *** = *p* < 0.001). Results are represented as mean percentages (±SEM) compared to conditions in which cells were stimulated with CXCL9, CXCL10 or CXCL11 without GAG. Percentages are means of 3–8 independent experiments.

Chemokine-induced CXCR3-signaling is associated with the release of intracellular calcium from the endoplasmic reticulum. Consequently, we reasoned that measuring the [Ca^2+^]_i_ after stimulation of cells with CXCL9, CXCL10 or CXCL11 with or without GAGs, would provide us with new insights on the effect of GAGs on the chemokine-induced G protein-dependent signaling through CXCR3. To this end, Chinese Hamster Ovarian (CHO) cells, stably transfected with CXCR3A and loaded with the calcium-binding fluorescent dye Fura-2, were stimulated with final concentrations of 3 ng/mL to 1 µg/mL CXCL9, CXCL10 or CXCL11. The corresponding chemokine-induced calcium responses were calculated using the Grynkiewicz equation. For CXCL10 and CXCL11, a concentration of 3 ng/mL resulted in an increase of the [Ca^2+^]_i_ with 106.8 nM (*n* = 39) and 304.5 nM (*n* = 18), respectively, and 3 ng/mL CXCL10 or CXCL11 was selected for further experiments in combination with GAGs. Cells were treated with CXCL10 or CXCL11 with or without 0.04 µg/mL, 2 µg/mL or 10 µg/mL GAG. Representative experiments are shown in Figure 3. The observed calcium responses were calculated as percentages of the corresponding reference values in the absence of GAGs. A dose-dependent negative correlation was found between the GAG concentration and the ability of CXCL10 and CXCL11 to evoke an intracellular calcium release through CXCR3 (Figure 4A,B). Heparin molecules with different length were tested in combination with CXCL10 and the longer heparin molecules were more potent inhibitors of the calcium response compared to the shorter DP8 form. For the less potent CXCL9, a concentration of 1 µg/mL was selected, resulting in an increase of the [Ca^2+^]_i_ with 598.1 nM (*n* = 4). Heparan sulfate also dose dependently inhibited the calcium response induced by this weaker CXCR3 ligand (Figure 4C). It remains to be elucidated whether the effect of GAGs on calcium signaling is due to direct binding of GAGs to chemokines, CXCR3 or both. In addition, it cannot be excluded that GAGs directly interfere with intracellular signaling. However, as expected, GAGs did not induce an increase of the [Ca^2+^]_i_ in the absence of chemokine (data not shown).

### 2.4. Effect of Soluble GAGs on CXCL10-Mediated CD26-Positive T Cell Chemotaxis In Vitro

Activated T cells express membrane-bound CD26. Thus, we wondered whether the observed GAG-mediated protection of CXCL10 against inactivation by CD26 was reflected in an increased CXCL10-induced T cell chemotaxis. To this end, we first confirmed CD26 expression and activity on cultured T cells. Flow cytometry was performed and revealed that, as expected, the majority of cultured cells expressed CD26 in addition to CD3 and CXCR3 (data not shown). Different cell concentrations were incubated with the CD26 substrate Gly-Pro-pNA and the enzymatic activity was determined. As expected, an increased enzymatic activity was found with increasing concentrations of T cells (data not shown). Purified soluble natural seminal fluid-derived CD26 was used as a positive control.

**Figure 5:**
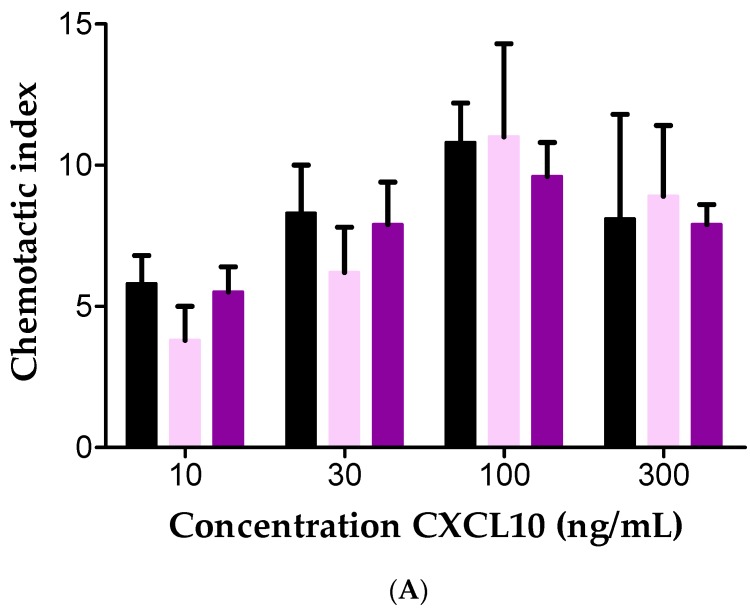

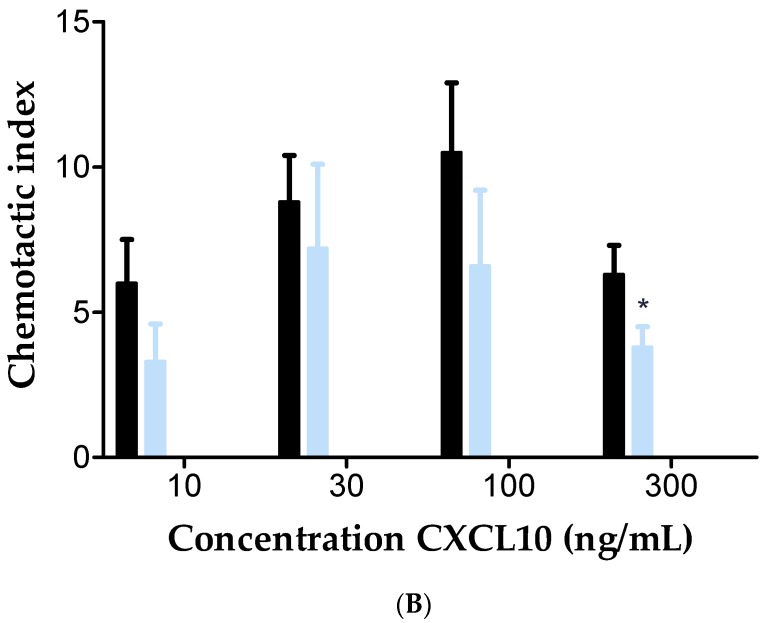
Effect of GAGs on CXCL10-induced T cell chemotaxis in vitro. The effects of: 0.04 µg/mL (light violet, ●), or 4 µg/mL (violet, ●) soluble heparan sulfate (**A**); or 4 µg/mL (light blue, ●) heparin (**B**) on the in vitro migratory response of PHA-activated T cells towards10–300 ng/mL CXCL10 was investigated using the 48 well Boyden chamber chemotaxis assay. Values represent the mean chemotactic index (±SEM) (*n* = 7). Statistical analysis was performed using the Mann-Whitney U test. * = *p* < 0.05; compared to stimulation with CXCL10 without GAG (black, ●).

Subsequently, in vitro Boyden chamber assays were conducted to evaluate the effects of GAGs on CXCL10-mediated chemotaxis of T cells. We investigated the migratory response of T cells towards 3 to 300 ng/mL CXCL10 with or without 0.04 or 4 µg/mL soluble heparin or heparan sulfate (Figure 5). Chemotactic indices were calculated by dividing the number of migrated cells to chemokine by the number of cells that migrated in response to buffer. As expected, a dose-dependent CXCL10-mediated T cell chemotaxis was observed with 10 ng/mL to 300 ng/mL CXCL10. In contrast to our expectations, presence of soluble heparan sulfate did not significantly affect CXCL10-induced T cell chemotaxis in vitro (Figure 5A). Heparin (4 µg/mL) significantly inhibited T cell chemotaxis to 300 ng/mL CXCL10 and a trend towards reduced chemotaxis was found for other CXCL10 concentrations (Figure 5B). It remains to be determined whether the lack of inhibition with heparan sulfate was due to in vitro counteraction between the two observed GAG-mediated phenomena, i.e. heparan sulfate-mediated protection of CXCL10 against processing by CD26 on the one hand, and the negative effect of heparan sulfate on the CXCR3-CXCL10 dialog on the other hand.

### 2.5. Inhibition of Membrane-Bound CD26 Did Not Affect CXCL10-Mediated T Cell Chemotaxis In Vitro

**Figure 6:**
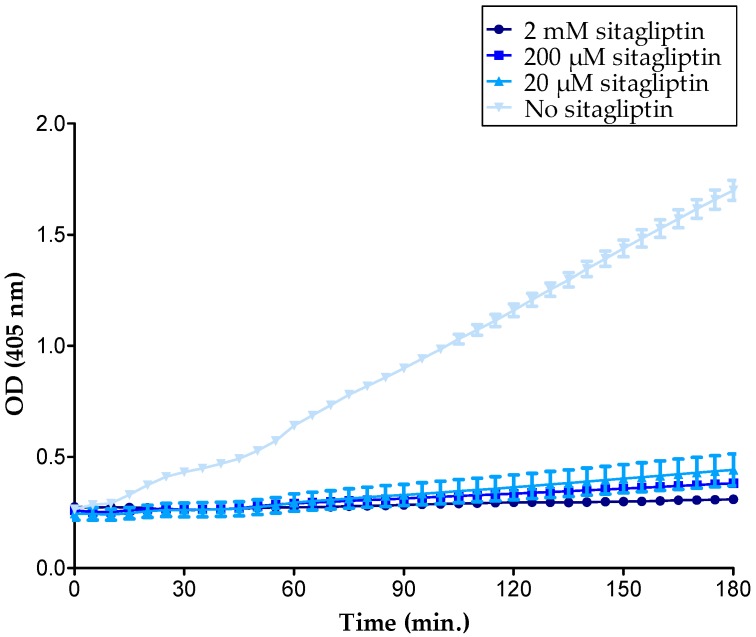
Sitagliptin inhibits CD26 proteolytic activity on T cells. In total, 3 × 10^6^ cells per mL were incubated with 500 µM Gly-Pro-pNA with or without 20 µM to 2 mM sitagliptin in 75 mM Tris-HCl buffer (pH 8.3). OD values were measured at 405 nm and plotted in function of time to construct CD26 activity curves. The figure shows mean values (±SEM) of a representative experiment.

**Figure 7:**
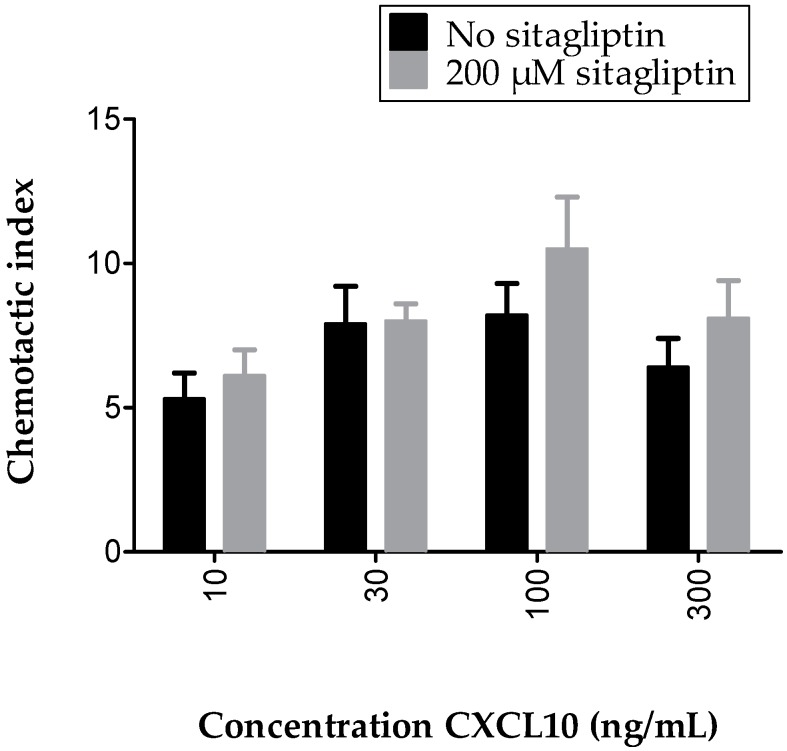
Sitagliptin did not affect CXCL10-induced T cell chemotaxis in vitro. The effects of treatment with 200 µM sitagliptin on the in vitro migratory response of PHA-activated T cells towards 10–300 ng/mL CXCL10 was investigated using the 48 well Boyden chamber chemotaxis assay. Values represent the mean chemotactic index (±SEM) (*n* = 6). Statistical analysis was performed using the Mann-Whitney U test.

To directly investigate the effects of specific inhibition of CD26-mediated cleavage of CXCL10 on CXCL10-directed T cell migration, chemotaxis assays were performed in the presence of the specific CD26 inhibitor sitagliptin. We first conducted a CD26 activity test with T cells that were treated with 0 to 2 mM sitagliptin and demonstrated that sitagliptin dose-dependently decreased the CD26 activity of cultured T cells (Figure 6). A concentration of 200 µM was selected for use in in vitro chemotaxis experiments. No significantly increased CXCL10-mediated T cell chemotaxis was observed when cells were treated with sitagliptin, which was in contrast with our expectations and with former in vivo studies [52,63] (Figure 7). However, in vivo, soluble CD26 is present on capillary endothelial cells and in body fluids including plasma, whereas, in our in vitro chemotaxis experiments, CXCL10 was only confronted with membrane-bound CD26 on the T cells. Therefore, it could be speculated that CXCL10 in in vitro assays was already bound to CXCR3 prior to potential truncation by CD26. Additionally, it cannot be excluded that, under the usedin vitro conditions, the amount of membrane-bound CD26 was too low to be able to detect a significant influence of its inhibition by sitagliptin.

### 2.6. Inhibition of CD26 Significantly Increased CXCL10-Induced Lymphocyte Influx into the Joint In Vivo

**Figure 8:**
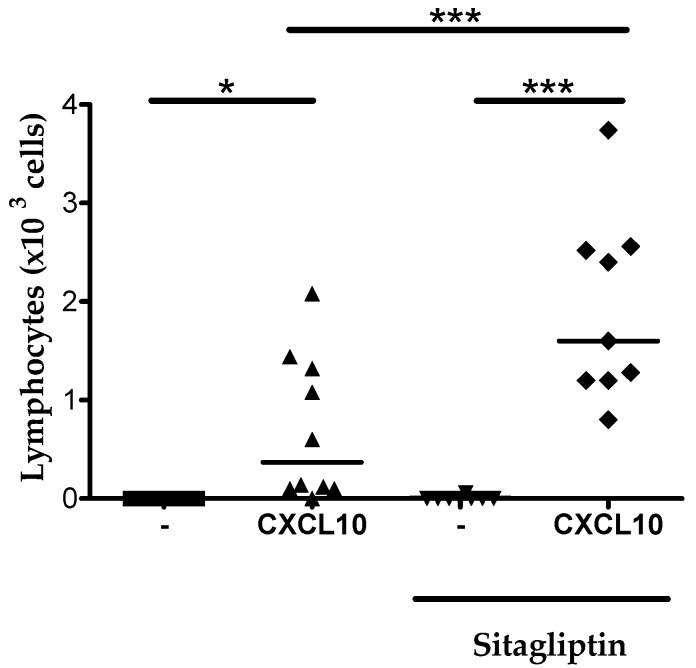
Sitagliptin-treatment of mice enhances CXCL10-mediated lymphocyte extravasation into the joint. The effect of CD26 inhibition on CXCL10-induced lymphocyte extravasation was determined in vivo. Vehicle or CXCL10 were injected into the tibiofemoral articulation of sitagliptin-treated C57BL/6 mice. Total leukocyte numbers migrated into the joint were determined 3 h post injection. Percentages of lymphocytes were differentially counted on May–Grünwald–Giemsa stained cytospins and were used to calculate total lymphocyte numbers. Each symbol represents an individual mouse (*n* ≥ 8). Horizontal lines indicate the median number of lymphocytes for each treatment group. Statistical analysis was performed using the Mann-Whitney *U* test. * *p* < 0.05, *** *p* < 0.001.

Chemokine injection into the tibiofemoral articulation was used as an experimental model to study the effect of specific CD26 inhibition in vivo, since this model has the advantage of very low basal leukocyte counts upon vehicle injection (Figure 8). Moreover, CXCL10 is typically a chemokine found in high concentrations in synovial fluids from inflammatory joints of patients with septic, rheumatoid or psoriatic arthritis [22,23]. To investigate the in vivo effect of CD26 inhibition on CXCL10-induced lymphocyte recruitment into the joint, CXCL10 was injected into the tibiofemoral articulation of sitagliptin-treated and untreated mice.

No lymphocyte infiltration was seen in mice that did receive vehicle injection in the joint. Injection of CXCL10 resulted in a lymphocyte recruitment in both untreated and sitagliptin treated mice (Figure 8). However, the number of lymphocytes in joints of mice that had received sitagliptin was significantly higher than the lymphocyte counts in mice that were not treated with the CD26 inhibitor. These results provide direct in vivo evidence that CD26 inhibition protects CXCL10 against cleavage, which is reflected into an enhanced lymphocyte extravasation into the joint. Moreover, these findings are in line with previous studies that reported that sitagliptin treatment in mice is translated into increased CXCL10-mediated lymphocyte infiltration into tumor tissue [63] and increased lymphocyte recruitment to intraperitoneally injected CXCL10 [52].

## 3. Discussion

Binding of chemokines to GAGs is essential to generate chemotactic gradients in vivo [32,33,34,35,36,37,38,39]. Moreover, it has been evidenced that the impact of the interaction with GAGs extends beyond the mere facilitation of chemokine retention on the endothelium, thereby locally concentrating chemokines on the cell surface [44]. Interaction with GAGs was found to mediate chemokine oligomerization and to provoke protection against proteolysis by specific enzymes. For example, CXCL12 was protected by heparan sulfate and heparin oligosaccharides against cleavage by CD26 [46]. CXCL12 or stromal derived factor 1 (SDF-1) is an ELR negative CXC chemokine that induces chemotaxis of lymphocytes and CD34 positive progenitor cells through activation of CXCR4. Via interaction with the same receptor, CXCL12 holds anti-HIV properties [64,65,66,67]. The chemotactic, CXCR4 dependent signaling and antiviral effects of CXCL12 are lost upon truncation by CD26 [68,69]. In the present study, we demonstrated that GAGs also protect CXCL9, CXCL10 and CXCL11 against proteolytic processing by CD26. Of note, the study by Sadir et al. [46] relied on colon carcinoma cells as source of CD26 activity whereas in our experiments, natural soluble CD26, isolated from human seminal fluid and purified to homogeneity, was used.

Another study reported that murine CCL11, also named eotaxin, is no longer processed by plasmin, elastase and cathepsin G upon interaction with immobilized heparin [47]. Here, the authors found that heparin directly inhibits the enzymatic activity of elastase and cathepsin G but not plasmin. In the present study, no evidence was found for GAGs to directly block the enzymatic activity of CD26. These results are in line with the aforementioned CXCL12 study were the investigators showed that the enzymatic activity of CD26 was not blocked by heparan sulfate [46]. We therefore suppose that the observed role for GAGs in offering the CXCR3 ligands protection against processing by CD26 is rooted at the level of chemokine–GAG interactions, thereby providing steric hindrance at the level of the NH_2_-terminal domain of the chemokine. For CXCL12 it was shown that the protection of the NH_2_-terminus depends on GAG-induced oligomerization of the chemokine rather than straight interaction with the NH_2_-terminal Lys residue [70]. It remains to be elucidated whether the protective effect of GAG interaction on CXCR3 ligands is a result of GAG-mediated chemokine oligomerization or direct steric hindrance at their NH_2_-terminus. Nota bene, GAG-mediated chemokine oligomerization seems an intriguing mechanism on its own, but the molecular details remain largely unknown. Indeed, although chemokine monomers seem responsible for GPCR activation, GAG-mediated induction or stabilization of chemokine oligomers is a prerequisite for the in vivo activity of certain chemokines including CXCL10 [33,36,71]. In return, oligomerization may enhance chemokine affinity for GAGs, which is also dependent on specific GAG density [72]. Oligomeric forms were described for CXCL9 and CXCL10 at physiological concentrations, whereas the oligomeric state of CXCL11 is less understood [36,73,74,75]. However, a key role for oligomerization of several chemokines including CXCL11 was recently demonstrated in facilitating reorganization and bridging of GAG chains, thereby conferring another level of complexity to the chemokine–GAG dialog [72].

The CXCR3 agonists, specifically CXCL9, CXCL10 and CXCL11, all contain a proline residue in the penultimate NH_2_-terminal sequence and are biologically relevant CD26 substrates. Upon incubation of 5 µM chemokine with a normal plasma concentration of 25 U/L CD26, the half-lives of CXCL10 and CXCL11 were previously demonstrated to be, respectively, not more than 4 and 2 min, whereas CD26-mediated cleavage of CXCL9 was found to occur less efficiently (half-life of 24 min under the same experimental conditions) [62]. Following cleavage by CD26, the three CXCR3 agonists are biologically inactive in chemotaxis and CXCR3-dependent signaling assays and show a decreased effect on T cells. Moreover, CXCL10(3–77) and CXCL11(3–73) act as CXCR3 antagonists [53]. The CD26-truncated isoform CXCL10(3–77) was originally isolated from natural sources including conditioned medium from MG-63 osteosarcoma cells and fibroblasts [22,58]. Studies with CXCL10^−/−^ mice established a crucial role for CXCL10 in the generation of effector T cells and in T cell trafficking in general [76]. This important physiological role of CXCL10, but also CXCL9 and CXCL11, combined with the fact that CXCL9(3–103), CXCL10(3–77) and CXCL11(3–73) are inactive, supports the idea that CD26-mediated proteolysis of the CXCR3 agonists may have major biological consequences. Results of multiple studies provided evidence in favor of this hypothesis. In mice, truncation of CXCL10 by CD26 reduces the infiltration of T cells into tumor tissue [63] and towards intraperitoneally injected CXCL10 [52] and consequently impairs the natural antitumor immunity Administration of the CD26 inhibitor sitagliptin significantly improved the natural antitumor immunity of these mice and their response to existing immunotherapies. In human, the biologically inactive isoform CXCL10(3–77) was found in plasma from patients that suffer from chronical hepatitis C viral infections [77]. Moreover, two prospective studies recently confirmed the CD26-mediated processing of human CXCL10 in vivo, and provided direct evidence in favor of CD26 inhibition to preserve intact CXCL10 [78]. Although human CXCL11 activates CXCR3 with higher potency and is even more efficiently processed by CD26 than CXCL10, the CD26—CXCL11 axis has been studied to a lesser extend in an in vivo context. It is likely to assume that this can be at least partially explained by the fact that murine CXCL11, in contrast to its human counterpart, contains a methionine residue in its penultimate position and is therefore no substrate for CD26. Interestingly, natural human CXCL11(3–73) was previously isolated from IFN-γ stimulated keratinocytes [59,60,61]. In addition, murine CXCL9 is no CD26 substrate due to the leucine residue that occupies the penultimate position in its NH_2_-terminal amino acid sequence. However, in view of the efficient cleavage of all three human CXCR3 agonists, our observed GAG-mediated protection may be highly significant depending on the local conditions in specific human tissues. Indeed injection of CXCL10 in joints still induces limited lymphocyte migration (Figure 8), whereas injection of CXCL10 in the peritoneum did not attract lymphocytes [52].

In the present study, we found that soluble GAGs significantly reduce the calcium mobilizing capacities of CXCL9, CXCL10 and CXCL11 through CXCR3. Given the general idea that the NH_2_-terminal chemokine domain facilitates receptor interaction whereas the COOH-region is considered the major GAG-binding domain, this observation may seem somewhat unexpected. However, it was previously suggested that both interaction domains are not necessarily restricted to, respectively, the NH_2_- and COOH-terminus. The relevance of this hypothesis for CXCL10 for example, is supported by the fact that mutation of amino acids 20 to 24, 46 and 47 impairs both the heparin affinity of the chemokine and its binding and signalization through CXCR3 [79]. Furthermore, citrullination of the most NH_2_-terminal arginine in CXCL10 or CXCL11 reduced their interaction with heparin [80]. At this point it remains to be elucidated whether the negative effect of GAGs on the potency of CXCR3 agonists to induce intracellular calcium release results from competition between chemokines and GAGs for CXCR3 binding or is rooted at the level of signal transduction, where GAGs potentially exert an inhibitory effect either directly or indirectly. Noteworthy, former studies reported that soluble GAGs also significantly reduce the CXCL8, CCL2, CCL3 and CCL5 mediated intracellular calcium mobilization [81]. Specifically, the authors showed that GAGs, in a competitive fashion, bind to chemokine receptors CXCR1, CXCR2 and CCR1. To our notice, no calcium mobilization studies with GAGs, CXCR3 and its chemokine ligands have been conducted before, but we speculate that GAGs may also interact with CXCR3.

Membrane-bound CD26 is a lymphocyte surface marker that is expressed by activated T cells [82]. In our study, we confirmed that cultured T cells were indeed characterized by CD26 expression and we demonstrated that the expressed enzyme was enzymatically active. Combined with the fact that CXCL10(3–77) is biologically inactive and our observation that GAGs protect CXCL10 against cleavage by CD26, we reasoned that in the presence of GAGs, intact CXCL10 would be preserved, thus resulting in enhanced CXCL10-mediated T cell chemotaxis compared to a condition without GAG. However, we found no significant differences in CXCL10-mediated T cell chemotaxis in vitro in the presence of heparan sulfate and a moderately reduced migration in the presence of heparin. Possibly, this could be explained by the negative effect of GAGs on the CXCL10-CXCR3 dialog. Indeed, it appears that the reduced CXCL10(3–77)-CXCR3 interaction overwrites the inhibitory effect of GAGs on CD26-mediated truncation of intact CXCL10 towards inactive CXCL10(3–77). To investigate the effect of specific inhibition of CD26-mediated cleavage on CXCL10-induced T cell chemotaxis in vitro, we evaluated the effect of administration of the CD26 inhibitor sitagliptin. In contrast to our expectations, no significant increase in CXCL10-directed T cell migration was found in vitro when cells were treated with sitagliptin. However, it was previously demonstrated that sitagliptin treatment in mice resulted in enhanced infiltration of T cells into the peritoneum or in tumor tissue in vivo [52,63]. Moreover, in the present study, we showed that intra articular injection of CXCL10 in sitagliptin-treated mice significantly enhances lymphocyte recruitment to the joint compared to mice that did not receive the CD26 inhibitor. These observations are in line with the idea that specific CD26 inhibition protects CXCL10 from inactivation in vivo. Indeed, in contrast to what is the case in our in vitro experiments, soluble CD26 and membrane-bound CD26 on non-lymphoid cells such as certain endothelial cells, fibroblasts and epithelial cells is present in vivo in addition to T cell-associated membrane-bound CD26 [83]. Therefore, in in vitro chemotaxis experiments, CXCL10 was not hindered by soluble CD26, but could only be inactivated by CD26 when it directly contacted the membrane-bound enzyme. A possible explanation for the lack of a significant effect of sitagliptin-mediated CD26 inhibition on CXCL10-induced chemotaxis in vitro, consequently, could be that CXCL10 was already bound to its receptor prior to interaction with membrane-bound CD26 on T cells. Furthermore, the amount of T cells in our experiments was fixed whereas during inflammation in vivo, for example, the number of activated T cells and consequently the availability of membrane-bound CD26, may drastically increase. Additionally, although we confirmed the CD26 expression and activity on cultured T cells, the CD26 activity on these cells was rather low. Thus, one could speculate that the amount of cell-bound CD26 activity in our in vitro tests was too low.

## 4. Materials and Methods

### 4.1. Cells and Reagents

#### 4.1.1. Chemokines and CD26

Full length recombinant human chemokines CXCL9, CXCL10 and CXCL11 were purchased from PeproTech (Rocky Hill, NJ, USA). An Activo-P11 automated solid phase peptide synthesizer (Activotec, Cambridge, UK) was used to chemically synthesize CXCL10, based on *N*-(9-fluorenyl) methoxycarbonyl (Fmoc) chemistry as described previously [84]. To avoid synthesis problems due to its COOH-terminal proline, a H-Pro-2Cl-Trityl resin (Activotec) was used for synthesis of CXCL10. The synthesized chemokine was purified to homogeneity with reverse phase–high performance liquid chromatography using a Source 5-RPC column (4.6 × 150 mm; GE Healthcare, Uppsala, Sweden). An acetonitrile gradient in 0.1% (*v*/*v*) TFA was used for elution of the synthesized protein and 0.7% of the effluent was directly injected into an electrospray–ion trap mass spectrometer (Bruker AmaZon SL mass spectrometer; Bruker Daltonics, Bremen, Germany). Fractions containing homogenous CXCL10 were selected, pooled, evaporated and dissolved in ultrapure water. Following folding of purified CXCL10 according to the protocol described by Loos et al. [84], the identity of the chemokine was confirmed by ion trap mass spectrometry and automated NH_2_-terminal sequencing based on the principle of Edman degradation (Procise 491 cLC sequencer, Applied Biosystems, Foster City, CA, USA). In addition, SDS-PAGE and bicinchoninic acid (BCA) protein assays (Pierce, Woodland Hills, CA, USA) were used to determine protein concentrations and purity.

Natural human soluble CD26 was isolated from human seminal fluid and purified to homogeneity by anion exchange and affinity chromatography as described [85].

#### 4.1.2. Cells

Chinese hamster ovary cells, stably transfected with CXCR3A (CHO/CXCR3A cells), were a gift from M. Parmentier (Université Libre de Bruxelles, Brussels, Belgium) and were cultured in Ham’s F12 medium (Lonza, Basel, Switserland) enriched with 1 mM sodium pyruvate, 400 µg/mL geneticin and 10% (*v*/*v*) fetal calf serum (FCS; Gibco, Paisley, UK). Peripheral blood mononuclear cells (PBMCs) were isolated from buffy coats or from fresh blood after centrifugation (10 min., 20 °C, 218 g) in a density gradient (Pancoll human, 1,077 g/mL; PAN Biotech GmbH, Aidenbach, Germany). Isolated PBMCs were washed with phosphate buffered saline (PBS) and cultivated in “Roswell Park Memorial Institute” 1640 (RPMI1640) medium (Cambrex Corporation, East Rutherford, NJ, USA) complemented with 10% (*v*/*v*) FCS, 0.1% (*w*/*v*) NaHCO_3_ (Gibco) and 0.05% (*w*/*v*) gentamycin (Gibco). T cells were activated with 0.002% (*w*/*v*) phytoheamagglutinin L (PHA; Sigma-Aldrich, St. Louis, MO, USA) at 37 °C during 2–5 days. Activated T cells were stimulated with recombinant human IL-2 (Peprotech) in fresh medium every 2–3 days, and were used in experiments 10–20 days after PHA activation and 2 days after IL-2 stimulation.

### 4.2. Proteolytic Processing of Chemokines by CD26 In Vitro

The chemokines CXCL9, CXCL10 or CXCL11 (20 µg/mL) were incubated with 12.5 units per liter (U/L) natural human CD26, with or without 0.88 to 26.4 µg/mL heparin, heparin DP8, heparin DP30, heparan sulfate, dermatan sulfate (Iduron, Chechire, U.K.), chondroitin sulfate A or chondroitin sulfate C (Sigma-Aldrich) in 50 mM EDTA; 1 mM Tris buffer (pH 7.5) in a total volume of 25 µL in low-binding tubes (Eppendorf LoBind Tube 1,5 mL, Eppendorf AG, Hamburg, Germany). An overview of the GAGs used in experiments is provided in Table 2. Nota bene, “DP” refers to the number of disaccharides. After 2 h of incubation, enzymatic reactions were terminated by acidification up to 0.08% (*v*/*v*) TFA. Chemokines were extracted and desalted from total samples on C18 ZipTip pipet tips (Millipore Corporation), eluted with 50% (*v*/*v*) acetonitrile in 0.1% (*v*/*v*) TFA, and analyzed by mass spectrometry.

### 4.3. CD26 Activity Assays

In a flat bottom 96 well plate, (Greiner Bio-One, Kremsmünster, Austria), 5 U/L purified natural human CD26 was incubated with 500 µM of the substrate Gly-Pro-*p*-nitroanilide (Gly-Pro-pNA; Sigma-Aldrich), with or without GAG, in 0.22 µm-filtered 75 mM Tris-HCl buffer (pH 8.3). Evolution of the optic density (OD) at 405 nm was followed using a spectrophotometer (BioTek PowerWave XS, Winooski, VT, USA) and represented the kinetics of CD26-mediated enzymatic conversion of colorless Gly-Pro-pNA into yellowish pNA. OD measurements were performed at 37 °C every 5 min for 3 h. CD26 activity curves were constructed by plotting OD values against time. Slopes of activity curves reflected the number of converted substrate molecules per min and were used to calculate enzymatic activities in U/L using the Lambert-Beer law. CD26 activity assays were also used to investigate the CD26 activity of PHA-activated T cells. Briefly, 10^4^ to 3 × 10^6^ T cells per mL in 75 mM Tris-HCl buffer (pH 8.3), with or without 20 µM to 2 mM sitagliptin (Merck Sharpe & Dohme (MSD) Whitehouse Station, NJ, USA), were incubated with 500 µM Gly-Pro pNA and the same protocol was followed.

### 4.4. Calcium-Mobilization Assays

CHO/CXCR3A cells (10 × 10^6^ per mL) in Ham’s F12 medium (Lonza) containing 10% (*v*/*v*) FCS were treated with 2.5 µM of the fluorescent dye Fura-2AM (Invitrogen, Carlsbad, CA, USA), 0.01% (*w*/*v*) Pluronic-F127 (Sigma) and 125 µM Probenecid solution (ICN Biomedicals Inc., Aurora, OH, USA) for 30 min at room temperature. Cells were washed with Ham’s F12 medium containing 10% (*v*/*v*) FCS, centrifuged (10 min, 4 °C, 177 g) and suspended in pH 7.0 Ca^2+^ buffer (“Hanks Balanced Salt Solution” (HBSS; Invitrogen) containing Ca^2+^ and Mg^2+^ and complemented with 10 mM HEPES (Gibco) and 0.1% (*v*/*v*) FCS) enriched with 125 µM Probenecid. Cells were kept on ice, centrifuged (10 min, 4 °C, 177 g), and suspended in Ca^2+^ buffer with Probenecid at final concentrations of 10^6^ cells per mL. An LS50 B luminescence spectrometer (Perkin Elmer, Waltham, MA, USA) was used to measure fluorescence and intracellular Ca^2+^ concentrations ([Ca^2+^]_i_) were calculated using the Grynkiewicz equation [86]. For each individual test condition, 1.8 × 10^6^ cells were preheated for 10 min at 30 °C, followed by stimulation with 3 to 1000 ng/mL CXCL9, CXCL10 or CXCL11, with or without 0.04 µg/mL, 2 µg/mL or 10 µg/mL GAG. R_max_ and R_min_ values were determined via treatment of cells with 50 µM digitonin and 10 mM EGTA (Sigma-Aldrich) in 20 mM Tris (pH 8.5; Merck, Darmstadt, Germany), respectively. Results were analyzed with WinLab32 software (Perkin Elmer). Fluorescence intensities of unloaded cells (not treated with Fura-2AM) were used to correct results for auto-fluorescence intrinsic to CHO/CXCR3A cells.

### 4.5. In Vitro Chemotaxis Assays

In vitro, 48-well Boyden chamber cell migration assays were used to determine chemotaxis of PHA-activated T cells in response to CXCL10 with or without GAG [87]. Briefly, CXCL10 and T cells were diluted in HBSS buffer enriched with 0.5% (*v*/*v*) human serum albumin (HSA; Red Cross Blood transfusion center, Leuven, Belgium). Wells in the lower part of the chamber were filled with 10–300 ng/mL CXCL10 with or without 0.04 or 4 µg/mL GAG in a total volume of 30 µL per well. Buffer without chemokine or GAG was used as a negative control. The lower chamber was covered with a 5 µm polycarbonate membrane (Nuclepore Track-Etch Membrane, Whatman, Little Chalfont, UK) that was treated with 20 µg/mL fibronectin (Gibco) in PBS overnight. The upper part of the chamber was filled with 2 × 10^6^ T cells per mL in buffer (50 µL per well), with or without 200 µM sitagliptin. Following 3 h of incubation at 37 °C, membranes were fixed and stained (Hemacolor Solution I–III, Merck). For each individual test condition, numbers of migrated T cells were counted microscopically in 10 separate fields. Chemotactic indices were determined by dividing total numbers of T cells that migrated to the sample through the total number of cells that migrated in response to buffer alone.

### 4.6. In Vivo Cell Migration Assay

The effect of CD26 inhibition on lymphocyte attraction was determined after intra-articular (i.a.) injection of human CXCL10 as described by Janssens et al. [88]. Briefly, the drinking water of 8-week-old C57BL/6 mice was complemented with 1.7 mg/mL sitagliptin from 3 days prior to i.a. injection of 1 µg synthetic full length CXCL10 diluted in 0.9% (*w*/*v*) NaCl. A *Limulus* amoebocyte lysate assay (Cambrex) demonstrated that endotoxin levels in injected samples were lower than 0.125 pg LPS per µg chemokine. During injection of 10 µL of the CXCL10 dilution, mice were anaesthetized using 3.75% (*w*/*v*) ketamine plus 0.25% (*w*/*v*) xylazine in PBS. Mice were sacrificed after 3 h of incubation, and articular cavities were washed with 3% (*w*/*v*) BSA in PBS. Total leukocyte numbers were calculated after staining with Turk’s solution in a Neubauer chamber, followed by differential counting of samples on cytospins that were stained with May–Grünwald–Giemsa. All in vivo experiments were conducted in the animal research facility of the University of Minas Gerais after approval by the Animal Ethical Committee.

### 4.7. Flow Cytometry

Flow cytometry was used to confirm the CXCR3 and CD26 expression of cultivated T cells. Tubes were filled with 3 × 10^5^ cells and centrifuged (5 min, 4 °C, 315 g). Supernatant was removed and cells were suspended in PBS containing Fc block (MACS Miltenyi Biotec, Bergisch Gladbach, Germany) and Aqua Zombie-BV510 (Biolegend, San Diego, CA, USA), according to the recommendations of the companies. Following 15 min of incubation at 20 °C, cells were centrifuged (5 min, 4 °C, 315 g) and pellets were suspended in flow cytometry buffer (PBS containing 2% (*v*/*v*) FCS and 2 mM EDTA). Samples were centrifuged (5 min, 4 °C, 315 g) and cells were diluted in fresh buffer. For each sample, recommended amounts of BV421-labeled anti-human CXCR3, PE-labeled anti-human CD3, PerCP-Cy5.5-labeled anti-human CD14, FITC-labeled anti-human CD26 (BD Pharmingen, San Diego, CA, USA) or APC-labeled anti-human CD14 (Biolegend), were added. Combined stainings were conducted with anti-CD3-PE, anti-CXCR3-BV421, anti-CD26-FITC and anti-CD14-PerCP-Cy5.5; anti-CD3-PE, anti-CXCR3-BV421 and anti-CD26-FITC; anti-CD3, anti-CD3-PE, anti-CXCR3-BV421 and anti-CD14-PerCP-Cy5.5 and finally with anti-CD3-PE, anti-CXCR3-BV421 and anti-CD14-APC. Isotype controls were performed to exclude non-specific binding. Untreated cells were used as negative controls. One sample of unstained cells was heated at 56 °C for 10–15 min to kill part of the cells, and served as a positive control for the Aqua Zombie staining. Antibody-treated cells were kept at 4 °C for 30 min, and were, respectively, suspended in flow cytometry buffer, centrifuged (5 min, 4 °C, 315 g) and decanted 3 times in a row. Resulting pellets were diluted in fixation buffer (flow cytometry buffer plus 0.04% (*w*/*v*) paraformaldehyde) and fluorescence intensities were determined with a BD LSRFORTESSA X-20 flow cytometer (BD Biosciences, Franklin Lakes, NJ, USA) equipped with 5 lasers. Results were analyzed with FlowJo software.

### 4.8. Statistics

Mann–Whitney U tests were performed to evaluate whether results of unpaired groups were significantly different or not. A *p* value of 0.05 or less was considered significant.

## Figures and Tables

**Table 1:** Effect of heparin on the proteolytic activity of CD26.

Concentration Heparin DP30 (µg/mL)	CD26 Activity (U/L)
0	4.6
10	4.19
100	4.35

**Table 2:** Specifications of GAGs used in experiments.

GAG	Source	Company	Composition	Relative Molecular Mass M_r_
Heparin	Porcine mucosa	Iduron	∆HexA,2S–GlcNS,6S–(IdoUA,2S–GlcNS,6S)n	±40 kDa ^a^
Heparin DP30	Porcine mucosa	Iduron	∆HexA,2S–GlcNS,6S–(IdoUA,2S–GlcNS,6S)_30_	>9 kDa ^b^
Heparin DP8	Porcine mucosa	Iduron	∆HexA,2S–GlcNS,6S–(IdoUA,2S–GlcNS,6S)_8_	±2.4 kDa ^b^
Heparan sulfate	Porcine mucosa	Iduron	GlcA-GlcNAc and IdoA/Glc-GlcNS (variable *O*-sulfation); contains both low and high sulfated heparan sulfates	±40 kDa ^a^
Dermatan sulfate	Porcine mucosa	Iduron	HexA-GalNAc,4S (88%); HexA-GalNAc (5%); HexA,2S-GalNAc,4S (7%)	±41 kDa ^b^
Chondroitin sulfate A	Bovine trachea	Sigma-Aldrich	Alternating Copoly β-glucuronic acid-(1→3)-*N*-acetyl-β-galactosamine-4-sulfate-(1→4)	±40 kDa ^a^
Chondroitin sulfate C	Shark cartilage	Sigma-Aldrich	Poly[β-glucuronic acid-(1→3)-*N*-acetyl-β-galactosamine-6-sulfate-(1→4)] alternating	±40 kDa ^a^

^a^ as experimentally determined by mass spectrometry; ^b^ according to the data sheet of the company.

## Supplementary Materials

Supplementary materials can be found at www.mdpi.com/1422-0067/18/7/1513/s1.

## Abbreviations

[Ca^2+^]_i_	Intracellular calcium concentration
GAG	glycosaminoglycan
GPCR	G protein-coupled receptor
M_r_	Relative molecular mass
