# Supplementary material for: Glycosaminoglycans Regulate CXCR3 Ligands at Distinct Levels: Protection against Processing by Dipeptidyl Peptidase IV/CD26 and Interference with Receptor Signaling
Source: Int J Mol Sci. 2017 Jul 13;18(7):1513. doi: 10.3390/ijms18071513 (PMC5536003; doi:10.3390/ijms18071513)
Supplement: Supplementary file 1 [file ijms-18-01513-s001.zip › ijms-205098-SI.pdf]

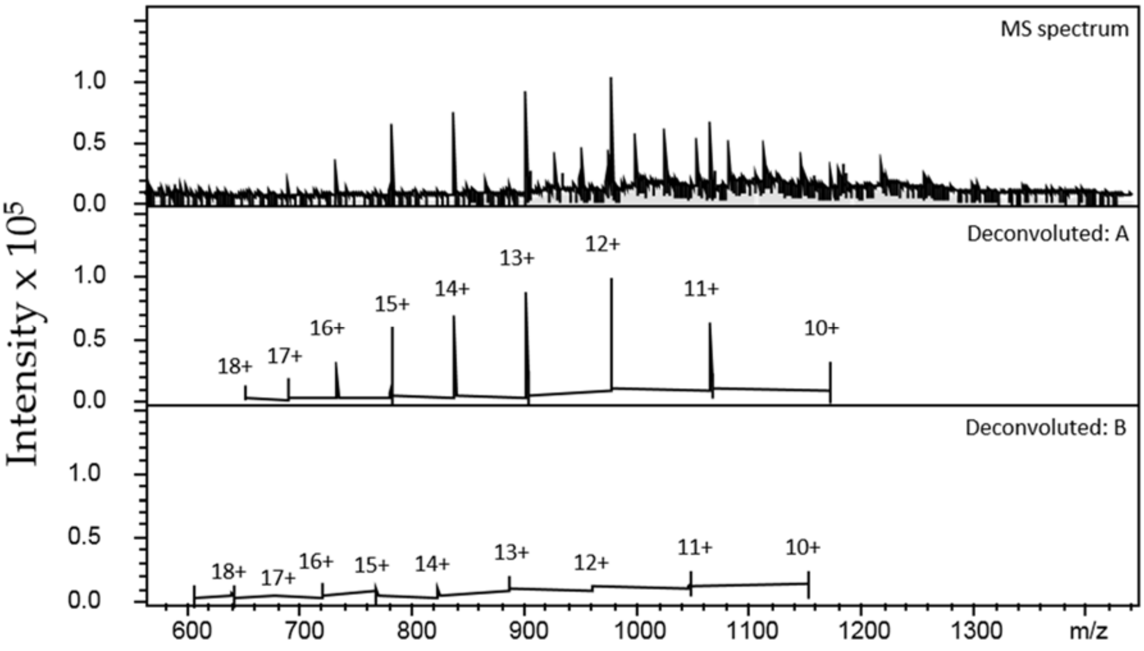

(a)

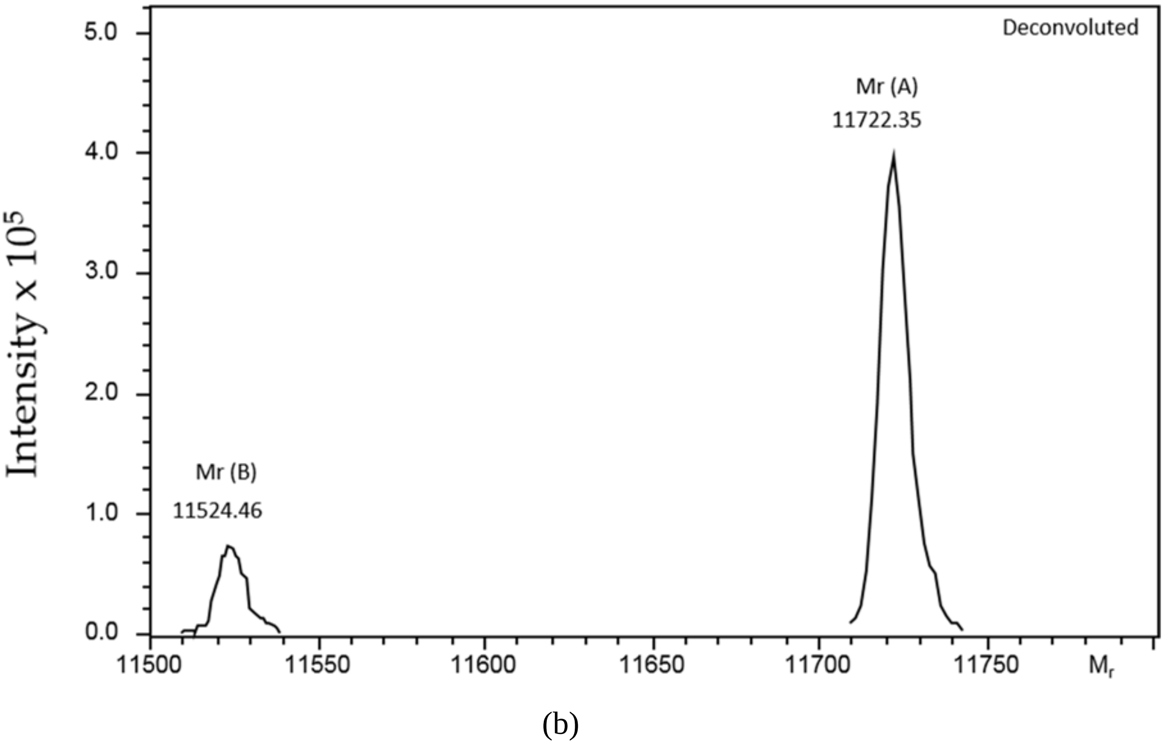

(b)

Figure S1. Mass spectrometry analysis of CXCL9 after incubation with CD26 and 2.64 µg/mL heparan sulfate. Recombinant human CXCL9 (20 µg/mL) was incubated with 12.5 U/L natural human CD26 and 2.64 µg/mL heparan sulfate in 50 mM Tris buffer supplemented with 1 mM EDTA (pH 7,5). The reaction was terminated after two hours by acidification to 0.08% (*v*/*v*) TFA and desalted on C18 ZipTips prior to analysis by mass spectrometry. The figure shows the raw mass spectrum (A, upper panel) and the specific multiple charged ions corresponding to intact CXCL9 (A, middle panel) and CXCL9 (3–

103) (A, lower panel). The deconvoluted mass spectrum shows the relative molecular masses of intact CXCL9 and CXCL9 (3–103) (panel B).

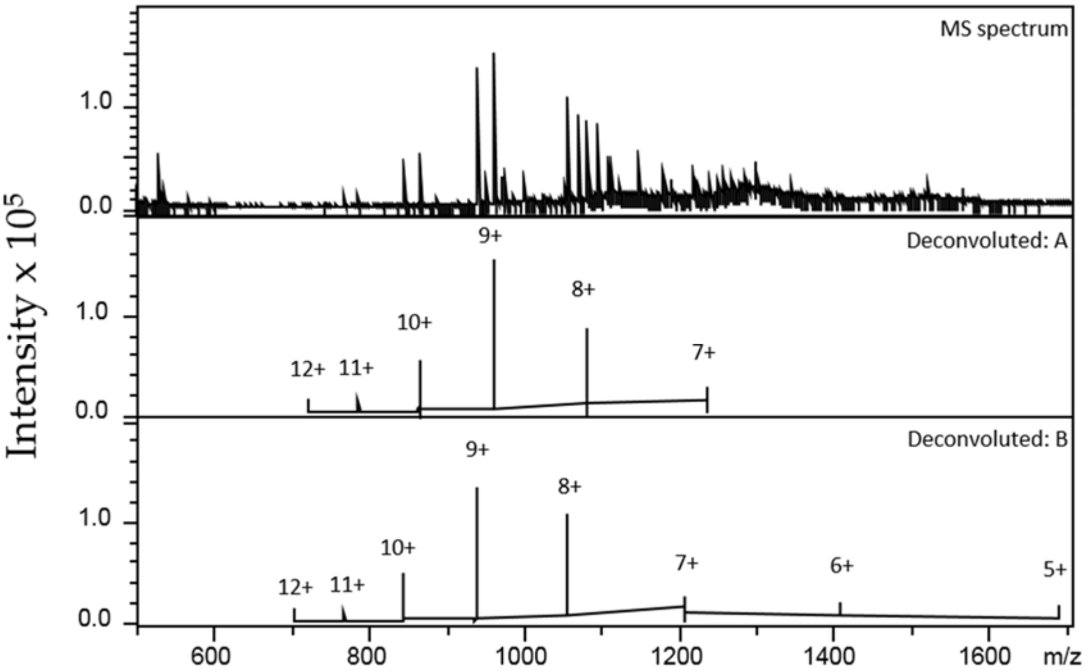

(a)

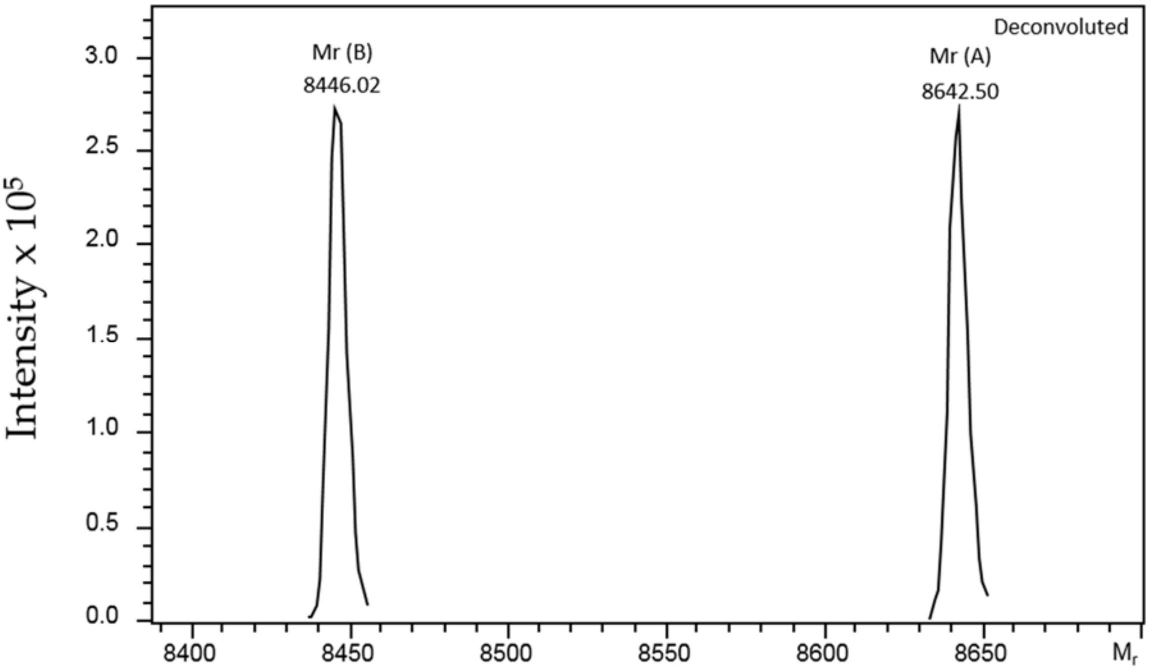

(b)

Figure S2. Mass spectrometry analysis of CXCL10 after incubation with CD26 and 2.64 µg/mL heparan sulfate. Recombinant human CXCL10 (20 µg/mL) was incubated with 12.5 U/L natural human CD26 and 2.64 µg/mL heparan sulfate in 50 mM Tris buffer supplemented with 1 mM EDTA (pH 7,5). The reaction was terminated after two hours by acidification to 0.08% (*v*/*v*) TFA and desalted on C18 ZipTips prior to analysis by mass spectrometry. The figure shows the raw mass spectrum (A, upper panel) and the specific multiple charged ions corresponding to intact CXCL10 (A, middle panel) and CXCL10 (3–

77) (A, lower panel). The deconvoluted mass spectrum shows the relative molecular masses of intact CXCL10 and CXCL10 (3–77) (panel B).

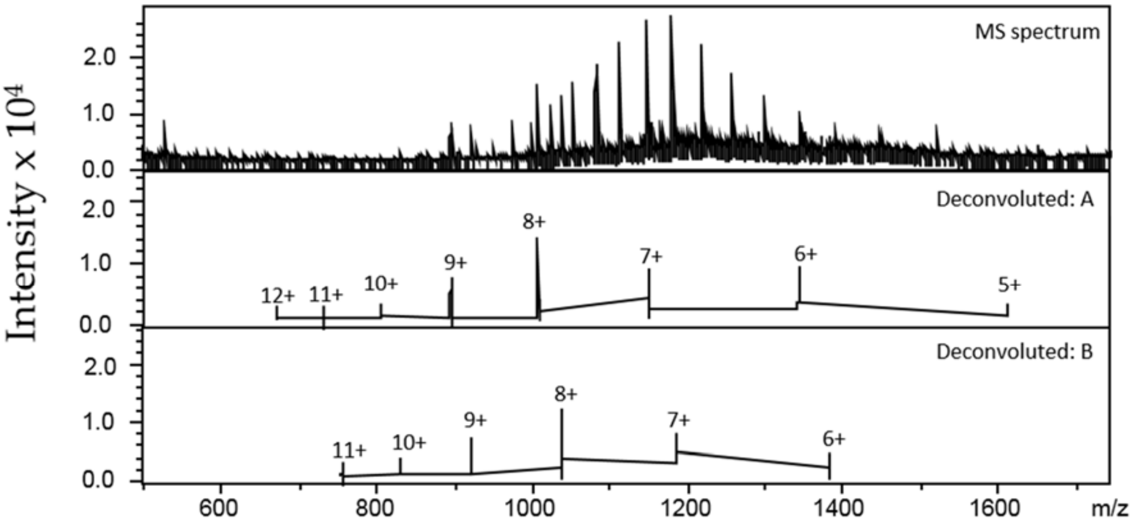

(a)

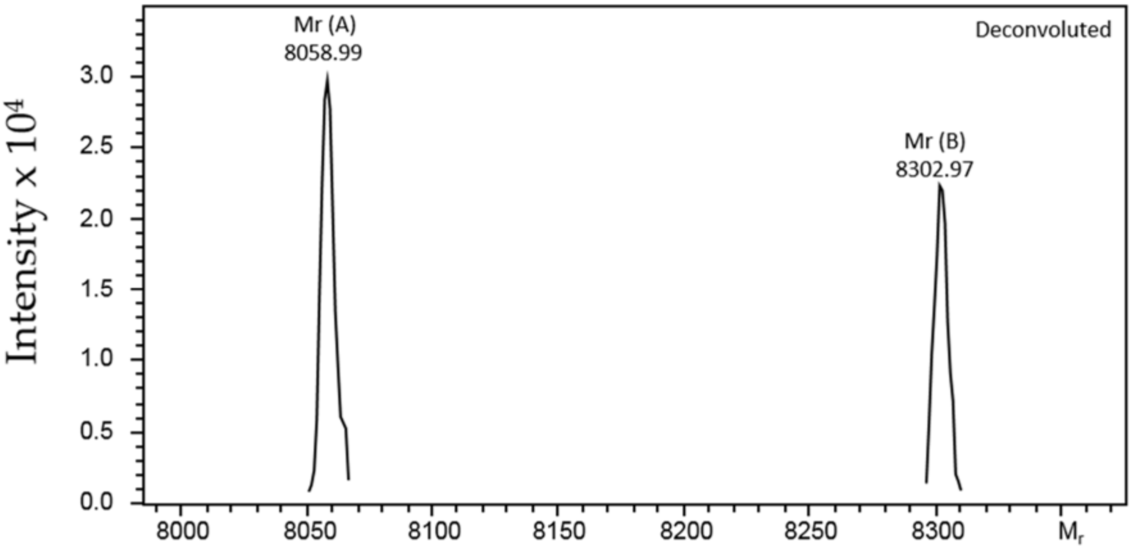

(b)

Figure S3. Mass spectrometry analysis of CXCL11 after incubation with CD26 and 2.64 µg/mL heparan sulfate. Recombinant human CXCL11 (20 µg/mL) was incubated with 12.5 U/L natural human CD26 and 2.64 µg/mL heparan sulfate in 50 mM Tris buffer supplemented with 1 mM EDTA (pH 7,5). The reaction was terminated after two hours by acidification to 0.08% (*v*/*v*) TFA and desalted on C18 ZipTips prior to analysis by mass spectrometry. The figure shows the raw mass spectrum (A, upper panel) and the specific multiple charged ions corresponding to CXCL11 (3–73) (A, middle panel) and intact

CXCL11 (A, lower panel). The deconvoluted mass spectrum shows the relative molecular masses of intact CXCL11 and CXCL11 (3–73) (panel B).
